# PMA Induces Vaccine Adjuvant Activity by the Modulation of TLR Signaling Pathway

**DOI:** 10.1155/2014/406514

**Published:** 2014-05-18

**Authors:** Dool-Ri Oh, Hu Won Kang, Jong-Ro Kim, Sunoh Kim, In-Kyu Park, Joon Haeng Rhee, Won Keun Oh, Young Ran Kim

**Affiliations:** ^1^Jeollanamdo Institute of Natural Resources Research, Jangheung, Jeollanamdo 529-851, Republic of Korea; ^2^Korea INSPharm Inc. 1065, Dong-myeon, Hwasun, Jeollanamdo 519-882, Republic of Korea; ^3^Department of Biomedical Science and BK21 PLUS Center for Creative Biomedical Scientists, Chonnam National University Medical School, Gwangju 501-757, Republic of Korea; ^4^Clinical Vaccine R&D Center, Chonnam National University Medical School, Gwangju 501-757, Republic of Korea; ^5^Korea Bioactive Natural Material Bank, Research Institute of Pharmaceutical Sciences, College of Pharmacy, Seoul National University, Seoul 151-742, Republic of Korea; ^6^Department of Pharmaceutical Engineering, Dongshin University, Naju, Jeollanamdo 520-724, Republic of Korea

## Abstract

Toll-like receptor (TLR) ligands are being developed for use as vaccine adjuvants and as immunomodulators because of their ability to stimulate innate and adaptive immune responses. Flagellin, a TLR5 ligand, was reported to show potent mucosal vaccine adjuvant activity. To identify ligands that potentiate the adjuvant activity of flagellin, we screened a plant library using HEK293T cells transiently cotransfected with phTLR5 and pNF-**κ**B-SEAP plasmids. The 90% EtOH extract from *Croton tiglium* showed significant NF-**κ**B transactivation in a TLR5-independent manner along with the increase of a flagellin activity. We have studied to characterize an active component from *Croton tiglium* and to elucidate the action mechanisms. Phorbol 12-myristate 13-acetate (PMA) was isolated as an active component of *Croton tiglium* by activity-guided fractionation, column chromatography, HPLC, NMR, and MS. PMA at a range of nM induced PKC-dependent NF-**κ**B activation and IL-8 production in both TLR5− and TLR5+ assay systems. In in vivo mouse vaccination model, PMA induced antigen-specific IgG and IgA antibody responses and increased IL-12 production corresponding to T cell responses in spleen lymphocytes. These results suggest that PMA would serve as an efficacious mucosal vaccine adjuvant.

## 1. Introduction

Toll-like receptors (TLRs) play crucial roles in innate immunity and can contribute to the development of appropriate adaptive immune responses [1, 2]. To date, 13 members of TLR family have been identified in mammals [3–5], and TLRs received special attention as potent adjuvant receptors [6]. Mammalian TLR5, expressed on epithelial cells and phagocytes such as dendritic cells and macrophages, recognizes flagellins of bacteria and subsequently activates the nuclear factor-kappa B (NF-*κ*B) pathway of host cells [7, 8]. We reported that a* Vibrio vulnificus* flagellin B (FlaB) showed strong mucosal vaccine adjuvant activity [9] and enhanced tumor-specific CD8^+^ T cell immune responses through TLR5 stimulation in a therapeutic cancer vaccine model [10]. Recently, FlaB combined with TNF*α* and IFN*α* was reported to generate potent dendritic cells which produce functionally active cytotoxic T lymphocytes [11].

Flagellin is a highly priced protein adjuvant candidate. To identify ligands that potentiate vaccine adjuvant activity of flagellin, we screened a plant extract library using HEK293T cells transiently cotransfected with phTLR5 and pNF-*κ*B-SEAP plasmids. The 90% EtOH extract from* Croton tiglium* L. (Euphorbiaceae) showed significant NF-*κ*B transactivation.* Croton tiglium* is a plant grown in tropical and subtropical zones, and the seed of* Croton tiglium* is well known as Ba-Dou (or Badou) in China and Korea. Ba-Dou has been used to treat gastrointestinal disorders, intestinal inflammation, rheumatism, headache, peptic ulcer, and visceral pain [12–14]. The sesquiterpenes and monoterpenes as the main components comprise the great parts of the extracted essential oil from seed. The toxic substances were found mainly in the bark and leaves of* Croton tiglium* and croton oil. In this study, we isolated phorbol 12-myristate 13-acetate (PMA) as an active component from* Croton tiglium* and investigated the action mechanisms in TLR signaling pathways.

## 2. Materials and Methods

### 2.1. Cell Culture

HEK293T and Caco-2 cells (ATCC, Manassas, VA) were cultured in Dulbecco modified Eagle medium (DMEM, WELGENE, Korea) supplemented with 10% fetal bovine serum (FBS, GIBCO, Invitrogen, Carlsbad, CA) at 37°C in a 5% CO_2_ incubator.

### 2.2. NF-*κ*B Reporter Assay

HEK293T cell is a human embryonic kidney cell line with SV40 large T-antigen for efficient transfection of plasmids. HEK293T cells seeded at 10^4^/well in 96-well plates were transfected with pNF-*κ*B-secreted alkaline phosphatase (pNF-*κ*B-SEAP, InvivoGen, San Diego, California), pIL-8-luciferase [9], or phTLR5 plasmids using Fugene 6 (Roche, Hague Road, Indianapolis). One day after transfection, the cells were replaced with fresh DMEM containing different concentrations of test agents for 1 day. NF-*κ*B-SEAP activities in cell culture supernatants were determined using QUANTI-Blue (InvivoGen) according to the manufacturer's instructions. Recombinant tag-free flagellin, FlaB, was purified as described elsewhere and contaminating lipopolysaccharide (LPS) was removed by using the AffinityPak Detoxi Gel endotoxin removing gel (Pierce Biotechnology, Inc., Rockford, IL) [9].

### 2.3. Plant Materials

The seeds of* Croton tiglium* were purchased from Chonnam Seangyack Nongob, Hwasun-gun, in April 2011, Republic of Korea. Plant sample was identified botanically by Professor Y. H. Moon. A voucher specimen (SNU2011-04) was deposited at the Herbarium of Seoul National University, Seoul, Republic of Korea.

### 2.4. Extraction and Isolation from the Seeds of* Croton tiglium*

The dried seeds of* Croton tiglium* (600 g) were extracted with 90% EtOH (2 L × 3 times) at room temperature. The combined 90% EtOH extract was then evaporated under reduced pressure using a rotary vacuum evaporator (EYELA, Japan). The dried crude extract of* Croton tiglium* (12 g) was suspended in water and divided successively with *n*-hexane (3 × 500 mL), CHCl_3_ (3 × 500 mL), EtOAc (3 × 500 mL), and *n*-BuOH (3 × 500 mL). The CHCl_3_ fraction (2.9 g), which showed strong enhanced activity on NF-*κ*B transcription, was chromatographed over a silica gel open column (5 × 40 cm; 63–200 *μ*m particle size, Merck, Darmstadt, Germany) eluting with gradient *n*-hexane/acetone (20 : 1, 10 : 1,…, 1 : 3, each 200 mL) to yield ten fractions (F1–F10) based on the TLC profile. Fraction F8 (1.05 g) was reapplied to an RP-C_18_ open column (4 × 30 cm; 40–63 *μ*m particle size) with a stepwise gradient of MeOH/H_2_O (1 : 2 to 10 : 1) to afford nine subfractions (F81–F89). Finally, subfraction F87 (30.2 mg) was purified by HPLC [OptimaPak C_18_ column (10  ×  250 mm, 10 *μ*m particle size, RS Tech, Korea); mobile phase MeOH in H_2_O containing 0.1% HCO_2_H (0–15 min: 85% MeOH, 15–40 min: 95% MeOH, 40–45 min: 100% MeOH)] to yield compound** 1** (*t*_*R*_ = 31.3 min, 5.2 mg) (Figure 2).

### 2.5. IL-8 ELISA in Caco-2 Cells

Caco-2 is a heterogeneous human epithelial colorectal adenocarcinoma cell and constituently expresses TLR5. Caco-2 cells were seeded at 5 × 10^4^/well in 48-well plates and were treated with ligands for 8 hours without FBS supplementation. IL-8 in the supernatant was measured by an ELISA kit (BioSource International, Inc., California, USA) according to the manufacturer's instructions.

### 2.6. Cell Staining and Fluorescence Microscopy

HEK293T cells in 8-well glass chamber plate (Nalge Nunc International, Rochester, NY) were transfected with phTLR5 using Fugene 6 (Roche). The cell culture was replaced with fresh DMEM containing PMA for 6 hours. After fixation for 15 minutes with 3.7% paraformaldehyde, the cells were rendered permeable by incubation in PBS with 0.2% Triton X-100 for 10 minutes. NF-*κ*B p65 protein was detected by immunostaining using a specific antibody (Santa Cruz Biotechnology, Delaware Avenue, Santa Cruz, CA) and Alexa Fluor-488-conjugated anti-rabbit-IgG antibody (Molecular Probes, Invitrogen, Eugene, OR). Fluorescence images were acquired using a fluorescence microscope (DXM1200C, Nikon).

### 2.7. Inhibition Assay of Pharmacological Antagonists on PMA-Induced NF-*κ*B Activity

To study the action mechanism, various pharmacological inhibitors were tested on PMA-mediated NF-*κ*B activation. Pharmacological inhibitors were used such as Wortmannin, Bay 11-7082, Genistein, GF109203X, PD98059, SB203580, SP600125, and U-73122 (Cell Signaling Technology, Danvers, MA) for the inhibition of phosphoinositide 3-kinase (PI3K), IkB-*α* phosphorylation, protein tyrosine kinase (PTK), protein kinase C (PKC), MEK1, SAPK2 (p38), jun N-terminal kinase (JNK), and phospholipase C (PLC), respectively.

### 2.8. Mice Immunization and ELISA

Five-week-old female BALB/c mice were intranasally immunized three times with 10 *μ*L of PBS containing oval albumin (OVA) as an antigen alone or in combination with PMA or FlaB at 7-day intervals. Seven days after the last immunization, feces and serum samples were collected from the immunized mice to assess antigen-specific antibody responses. All animal procedures were conducted in accordance with the guidelines of the Animal Care and Use Committee of Chonnam National University. OVA-specific antibodies were determined by ELISA followed by the methods as described elsewhere [9]. Absorbance was read by an ELISA microplate reader (Power Wavex340, NIO-TEK-INS TRUMENTS, INC) at 450 nm.

### 2.9. Cytokine Assay in Splenocytes from Vaccinated Mice

Spleen lymphocytes were prepared from the immunized mice by using lymphoprep according to the manufacturer's instructions (AXIS-SHIELD PoC AS, Norway). The lymphocytes were cultured in RPMI 1640 medium containing 10% FBS, 100 U/mL penicillin, and 100 *μ*g/mL streptomycin and incubated with OVA (10 *μ*g/mL) at 37°C for 2 days. The levels of interleukin 12 (IL-12) were measured by using sandwich ELISA kits following the manufacturer's experimental protocols (Biolegend, USA).

### 2.10. Statistical Analysis

All values are expressed as means ± standard error of the mean (SEM). Statistical comparisons were made using Student's *t*-test. All experiments were repeated three times and the results from a representative experiment were shown.

## 3. Results and Discussion

### 3.1. *Croton tiglium* and Its Chloroform Fraction Induced NF-*κ*B Transcription in TLR5-Independent Pathway and Enhanced FlaB Activity

**Figure 1:**
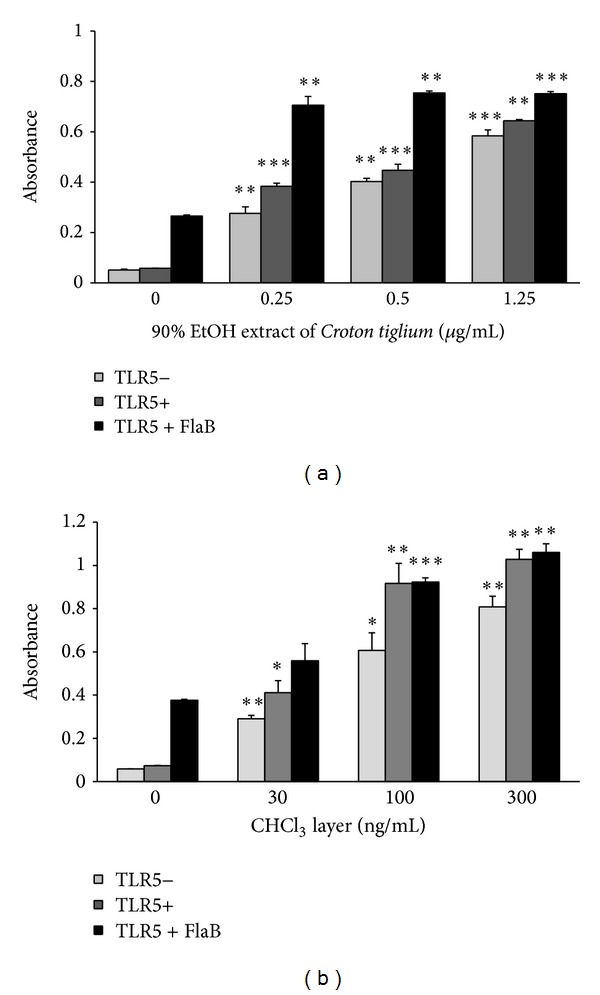
*Croton tiglium *and its chloroform fraction induced NF-*κ*B transcription in TLR5-independent manner and increased the FlaB activity. (a) 90% EtOH extract of* Croton tiglium* increased FlaB-mediated NF-*κ*B transcription. HEK293T cells transiently transfected with pNF-*κ*B-SEAP and phTLR5 were treated with FlaB and 90% EtOH extract of* Croton tiglium* for 1 day. SEAP activities were determined in the cell culture supernatants using QUANTI-Blue. 90% EtOH extract of* Croton tiglium* induced NF-*κ*B transcription in TLR5-independent manner and increased the FlaB activity. (b) The chloroform fraction of* Croton tiglium *increased NF-*κ*B transcription. The chloroform fraction of* Croton tiglium *increased significantly NF-*κ*B transcription in HEK293T cells transiently transfected with pNF-*κ*B-SEAP and phTLR5 regardless of TLR5. The data indicate the mean and SEM from three experiments (^∗^ *P* < 0.05, ^∗∗^ *P* < 0.01, ^∗∗∗^ *P* < 0.001).

TLR5 recognizes bacterial flagellin and activates a transcription factor NF-*κ*B hence inducing proinflammatory cytokine production in mammalian cells [7]. We studied to identify herbal medicines that enhanced NF-*κ*B activity of a flagellin, FlaB. TLR5 and NF-*κ*B-SEAP were overexpressed in HEK293T cells by transfection with the plasmids and a plant extract library was screened. The 90% EtOH extract from* Croton tiglium* increased NF-*κ*B transcription in HEK293T cells in TLR5-independent pathway and enhanced the FlaB activity inducing TLR5-dependent NF-*κ*B activation (Figure 1(a)). To identify active components of* Croton tiglium*, the 90% EtOH crude extract was divided into 5 fractions: *n*-hexane, chloroform, ethyl acetate, *n*-butanol, and water layers. The chloroform fraction showed a more significant effect than the other fractions on NF-*κ*B activity at concentrations of 30~300 ng/mL (Figure 1(b)).

### 3.2. Structure Determination and Identification of Active Component Inducing NF-*κ*B Activation from* Croton tiglium*

**Figure 2:**
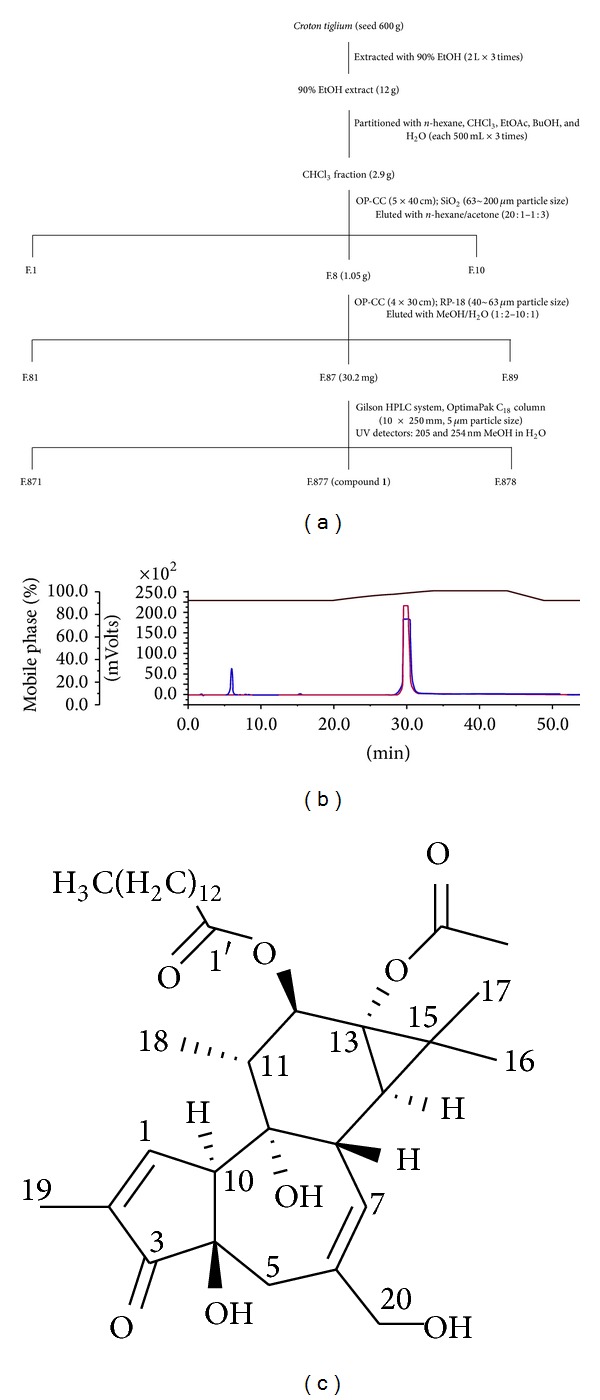
Isolation procedures of an active compound from* Croton tiglium.* (a) Column chromatography and HPLC.Components of the chloroform fraction from* Croton tiglium* were divided using column chromatography. The dried chloroform fraction was eluted on a silica gel column (5 × 40 cm; Merck, 63–200 *μ*m particle size) with a solvent gradient of hexane/acetone (20 : 1 to 1 : 3 ratios) to yield ten fractions (Fr.1–Fr.10). Fr.8 showing the most potent activity was divided into 9 subfractions (Fr.81–Fr.89) using RP-C_18_ column with a solvent gradient of MeOH/H_2_O (1 : 2 to 10 : 1). Fr.87 was applied to 8 fractions (Fr.871–Fr.878) using Gilson HPLC system with OptimaPak C_18_ column (10 × 250 mm, 5 *μ*m particle size). For activity-guided fractionation, pNF-*κ*B reporter activities of each fraction were evaluated in HEK293T cells transiently transfected with pNF-*κ*B-SEAP and phTLR5. (b) HPLC comparison of compound** 1** and PMA. An isolated compound** 1** from* Croton tiglium* was analyzed by coinjection with PMA standard from Sigma Co. (St. Louis, USA) by a Gilson HPLC with the 321-pumps systems; UV/Vis-155; 234-autoinjector; an OptimaPak C_18_ column (10 × 250 mm, particle size 5 *μ*m), using a gradient of methanol and 0.1% formic acid in H_2_O as mobile phase. Detection was analyzed with two channels at 205 and 254 nm (blue line; 205 nm, red line; 254 nm). Solvent elution was carried out with a gradient of methanol and 0.1% formic acid in H_2_O as mobile phase, at a flow rate of 2 mL/min. PMA and compound** 1** had the same retention time at 31.3 minute. (c) The chemical structure. The chemical structure of compound** 1** was confirmed by ^1^H and ^13^C NMR spectra (Varian Unity Inova 600 MHz spectrometer) and MS data (Micromass, Wythenshawe, UK).

In order to isolate an active component, the chloroform fraction from* Croton tiglium* extract was subjected to a succession of chromatographic procedures including silica gel chromatography, RP-C_18_, and HPLC (Figure 2(a)). Each fraction was tested on NF-**κ**B transcription in HEK293T cells and the activities were shown in Table 1. When phorbol 12-myristate-13-actate [PMA; synonym: 12-*O*-tetradecanoylphorbol-13-actate (TPA)] from Sigma Co. (St. Louis, USA) and an isolated compound (Compound** 1**) were coinjected into HPLC, the same retention time (*t*_*R*_) at 31.3 min suggested that it was an identical compound (Figure 2(b)). For further confirmation of the chemical structure of isolated compound, ^1^H and ^13^C NMR (nuclear magnetic resonance) spectra of 1 were measured on a Varian Unity Inova 600 MHz spectrometer at the Korea Basic Science Institute (KBSI, Gwangju Center, Korea). The ^1^H and ^13^C NMR spectroscopic data of Compound** 1** showed the characteristic signals for an *α*, *β*-unsaturated carbonyl group [(*δ*_H_ 7.57, 1H, br s, H-1; *δ*_C_ 160.7, C-1); *δ*_C_ 132.8, C-2; *δ*_C_ 208.8, C-3], a trisubstituted double bond [(*δ*_H_ 5.66, 1H, d, *J* = 4.6 Hz, H-7; *δ*_C_ 129.2, C-7); *δ*_C_ 140.4, C-6], an oxymethine (*δ*_H_ 5.39, 1H, d, *J* = 10.1 Hz, H-12; *δ*_C_ 76.5, C-12), and an oxymethylene [*δ*_H_ 4.01 and 4.00, AB 2H, *J* = 12.8 Hz, H_2_-20; *δ*_C_ 68.0, C-20] of a phorbol ester system [15, 16]. As the NMR and MS data [*m*/*z* 616.3980, Micromass QTOF2 (Micromass, Wythenshawe, UK)] are identical with those reported for PMA [16, 17], Compound** 1** was finally determined as PMA (Figure 2(c)).

*Compound* **1**. Colorless oil; ^1^H  (600 MHz, in CDCl_3_): *δ*_H_ 7.57 (1H, br s, H-1), 5.66 (1H, d, *J* = 4.6 Hz, H-7), 5.51 (1H, br s, OH-9), 5.39 (1H, d, *J* = 10.1 Hz, H-12), 4.01 and 4.00 (2H, AB peaks, *J* = 12.8 Hz, H_2_-20), 3.23 (1H, br s, H-10), 3.21 (1H, t, *J* = 5.5 Hz, H-8), 2.52 and 2.46 (2H, AB peaks, *J* = 19.3 Hz, H-5), 2.30 (2H, m, H-2′), 2.12 (1H, m, H-11), 2.07 (3H, s, acetyl), 1.76 (3H, dd, *J* = 2.7, 1.4 Hz, H-19), 1.60 (2H, m, H-3′), 1.18–1.31 [26, (4′–13′ methylene) and 2 × methyl (H-16 and H-17)], 1.06 (1H, d, *J* = 5.0 Hz, H-14), 0.87 (3H, d, *J* = 6.4 Hz, H-18), 0.86 (3H, t, *J* = 6.5 Hz, H-14′); ^13^C NMR data (150 MHz, in CDCl_3_): *δ*_C_ 160.7 (C-1), 132.8 (C-2), 208.8 (C-3), 73.7 (C-4), 38.6 (C-5), 140.4 (C-6), 129.2 (C-7), 39.1 (C-8), 78.1 (C-9), 56.2 (C-10), 42.9 (C-11), 76.5 (C-12), 65.6 (C-13), 36.2 (C-14), 25.6 (C-15), 23.8 (C-16), 16.8 (C-17), 14.1 (C-18), 10.1 (C-19), 68.0 (C-20), 173.7 (C-1′), 34.6 (C-2′), 25.2 (C-3′), 29.0–29.6 (C-4′ to C-11′), 31.9 (C-12′), 22.7 (C-13′), 14.1 (C-14′), 173.7 and 21.1 (acetyl); EIMS *m*/*z* 616.3980 (calcd for C_36_H_56_O_8_, 616.3975).

### 3.3. PMA Increased Significantly FlaB-Mediated NF-*κ*B Activity and IL-8 Production in TLR5-Independent Pathway

**Figure 3:**
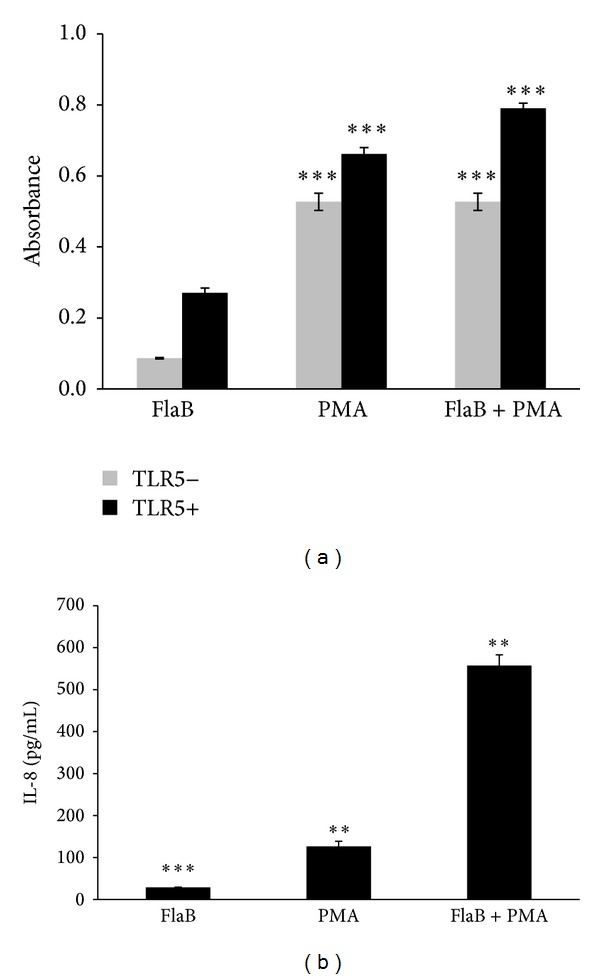
PMA induced NF-*κ*B transactivation and IL-8 production in TLR5-independent pathway. (a) PMA induced NF-*κ*B transactivation. HEK293T cells transfected with pNF-*κ*B-SEAP and phTLR5 plasmids were replaced with the fresh DMEM containing FlaB (20 ng/mL) or PMA (10 ng/mL) and incubated for 1 day. SEAP activity of the cell supernatants was determined in the supernatants using QUANTI-Blue. (b) PMA increased significantly FlaB-mediated IL-8 production in Caco-2 cells. Caco-2 cells were treated with PMA (100 ng/mL) with or without FlaB (1 *μ*g/mL) for 8 hours. IL-8 concentrations in the supernatant were determined by ELISA. PMA increased significantly FlaB-induced IL-8 production. The data indicate the mean and SEM from three experiments (^∗∗^ *P* < 0.01, ^∗∗∗^ *P* < 0.001).

PMA, an active component of* Croton tiglium*, was evaluated whether it could stimulate FlaB-mediated NF-*κ*B activity and IL-8 production. PMA at a low concentration of 10 ng/mL increased NF-*κ*B activity with or without FlaB (20 ng/mL) (Figure 3(a)). PMA also enhanced significantly FlaB-mediated IL-8 production in Caco-2 cells expressing TLR5 constitutively (Figure 3(b)). These results suggest that PMA is an active component of* Croton tiglium *in NF-*κ*B transactivation.

### 3.4. PMA Induced the Translocation of NF-*κ*B from Cytosol into Nucleus

**Figure 4:**
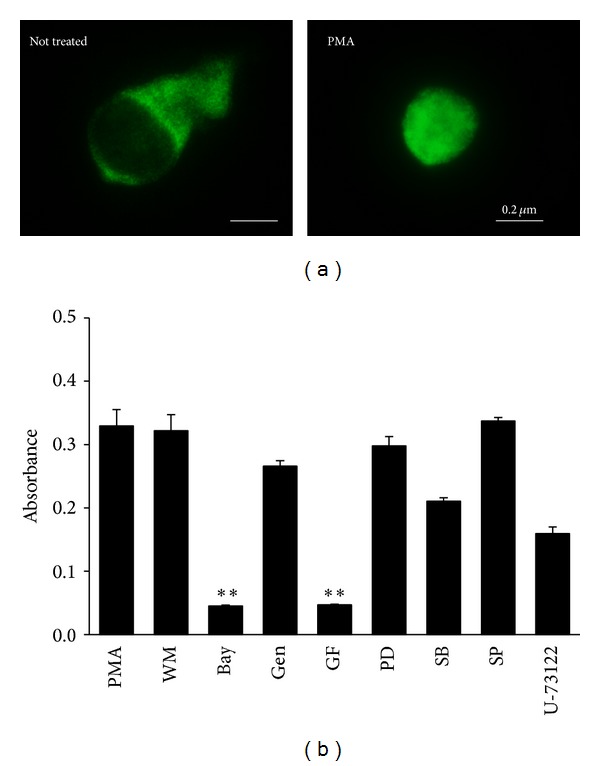
Action mechanism of PMA on TLR signaling pathway. (a) PMA induced nuclear translocation of NF-*κ*B p65 subunit. HEK293T cells were transfected with phTLR5 for 1 day and treated with or without PMA (10 ng/mL) for 6 hours. The cells were fixed, permeabilized, and immunostained with a polyclonal antibody against NF-*κ*B p65, followed by Alexa Fluor-488-conjugated anti-rabbit-IgG antibody. PMA induced nuclear translocation of NF-*κ*B p65 subunit in HEK293T cells. (b) PMA induced NF-*κ*B activation through PKC-dependent pathway. HEK293T cells transfected with pNF-*κ*B-SEAP and phTLR5 plasmids were treated with PMA (10 ng/mL) in the presence of pharmacological inhibitors. PMA-induced NF-*κ*B activity was blocked by the treatment of a PKC inhibitor (GF109203X) and an I*κ*B inhibitor (Bay11-7082). Wortmannin (WM), Bay 11–7082 (Bay), Genistein (Gen), GF109203X (GF), PD98059 (PD), SB203580 (SB), SP600125 (SP), and U-73122. The data indicate the mean and SEM from three experiments (^∗∗^ *P* < 0.01).

Like other members of the NF-**κ**B family, p65 resides in the cytoplasm in an inactive form bound to inhibitory I*κ*B protein. Cellular activation results in the nuclear translocation of p50 : p65 for initiating gene transcription. We therefore assessed the nuclear translocation of NF-*κ*B p65 subunit after its activation by PMA treatment (10 ng/mL) to the HEK293T cells by immunostaining. NF-*κ*B p65 was translocated from cytosol to nucleus after PMA treatment (Figure 4(a)).

### 3.5. A PKC Inhibitor Blocked PMA-Induced NF-*κ*B Activation in HEK293T Cells

To study the action mechanism of PMA, various pharmacological inhibitors were tested on PMA-mediated NF-*κ*B activation. Two hours after inhibitor incubation, the cells were treated with PMA at a concentration of 10 ng/mL. The supernatants were collected after 1 day of PMA treatment and NF-*κ*B-SEAP levels were determined by QUANTI-Blue. PMA-induced NF-*κ*B activation was blocked by the treatment of a PKC inhibitor GF109203X and an I*κ*B-*α* degradation inhibitor Bay11-7082 (Figure 4(b)). These results indicate that PMA induces NF-*κ*B activation through PKC-dependent I*κ*B phosphorylation.

### 3.6. PMA Increased OVA-Specific Systemic Immune Response in Mice

**Figure 5:**
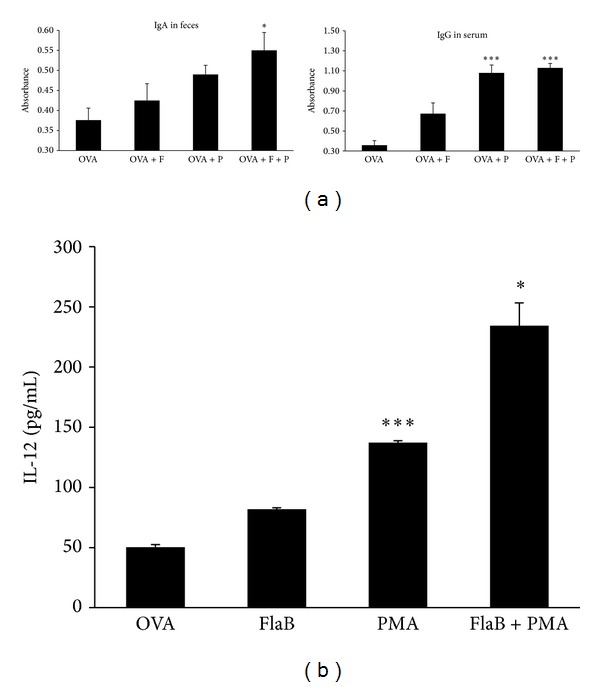
PMA potentiated FlaB vaccine adjuvant activity by the increase of IL-12 production. (a) PMA enhanced significantly OVA-specific IgA and IgG antibody responses. The BALB/c mice were intranasally immunized with OVA (100 *μ*g) alone or OVA in combination with FlaB (F) or PMA (P), three times in 7-day intervals. OVA-specific antibodies were measured by ELISA. Feces IgA and serum IgG levels were increased in OVA plus FlaB and PMA mice group (OVA+F+P). (b) PMA induced IL-12 production in mice spleen lymphocytes. Spleen lymphocytes from vaccinated mice were cultured with OVA (10 *μ*g/mL) at 37°C for 2 days. Cytokine levels were measured by sandwich ELISA kits following the manufacturer's experimental protocols. IL-12 was significantly increased in PMA combined mice splenocytes. The data indicate the mean and SEM from three experiments (^∗^ *P* < 0.05, ^∗∗∗^ *P* < 0.001).

BALB/c mice were intranasally immunized three times with antigen OVA alone or in combination with FlaB or PMA under anesthesia. Seven days after immunization, feces and serum samples were collected from the immunized mice to assess antigen-specific antibody responses. OVA-specific IgA and IgG antibodies were significantly increased by the administration of OVA plus PMA or FlaB (Figure 5(a)). These results suggest that PMA has vaccine adjuvant activity.

### 3.7. PMA Increased IL-12 Production in Spleen Lymphocytes of Vaccinated Mice

Spleen lymphocytes were prepared by using lymphoprep from vaccinated mice and cytokines were analyzed by ELISA. IL-12 production was significantly increased in spleen lymphocytes isolated from OVA-vaccinated mice treated with PMA (Figure 5(b)).

## 4. Conclusion and Discussion

The present study demonstrates the vaccine adjuvant effect of PMA isolated from* Croton tiglium*. To identify herbal medicines potentiating the vaccine adjuvant effect of flagellin, a plant library was screened using HEK293T cells transiently cotransfected with phTLR5 and pNF-*κ*B-SEAP plasmids. Because HEK293T cells do not express any TLRs, the cells transfected with phTLR5 plasmid DNA were used for TLR5-dependent response in the absence of other TLRs. Flagellin, a TLR5 agonist, activated a transcription factor NF-*κ*B only in the presence of TLR5 (Figure 1). In contrast, the 90% EtOH extract of* Croton tiglium* and its chloroform layer significantly stimulated NF-*κ*B transcription both in TLR5− and TLR5+ screening systems (Figure 1). PMA was isolated and characterized as an active component from* Croton tiglium* (Figure 2) by activity-guided fractionation, column chromatography, HPLC, NMR, and MS. PMA induced NF-*κ*B transactivation in a TLR5-independent manner and increased IL-8 production in Caco-2 cells constituently expressing some TLRs (Figure 3). These results indicate that PMA is not a specific ligand for TLR5. The effect of PMA was dependent on NF-*κ*B translocation and PKC activation (Figure 4(b)). Finally, PMA or FlaB enhanced antigen-specific IgG and IgA antibody responses in intranasally OVA-immunized mice (Figure 5(a)). In addition, PMA increased IL-12 production corresponding to T cell responses (Figure 5(b)). The present study suggests that PMA activating PKC of TLR signaling pathway has a possibility for being an efficacious mucosal vaccine adjuvant.

## Figures and Tables

**Table 1:** Effects of fractions from *Croton tiglium* on NF-*κ*B transactivation.

Fractions (10 ng/mL)	Absorbance	Fractions (3 ng/mL)	Absorbance	Fractions (1 ng/mL)	Absorbance
Fr.1	0.084 ± 0.004	Fr.81	0.121 ± 0.002	Fr.871	0.080 ± 0.006
Fr.2	0.094 ± 0.008	Fr.82	0.132 ± 0.003	Fr.872	0.082 ± 0.004
Fr.3	0.086 ± 0.004	Fr.83	0.121 ± 0.003	Fr.873	0.120 ± 0.008
Fr.4	0.071 ± 0.004	Fr.84	0.124 ± 0.006	Fr.874	0.092 ± 0.004
Fr.5	0.112 ± 0.001	Fr.85	0.125 ± 0.004	Fr.875	0.077 ± 0.004
Fr.6	0.121 ± 0.002	Fr.86	0.223 ± 0.008	Fr.876	0.103 ± 0.000
Fr.7	0.975 ± 0.086	**Fr.87**	0.981 ± 0.035	**Fr.877**	0.318 ± 0.086
**Fr.8**	0.991 ± 0.026	Fr.88	0.631 ± 0.016	Fr.878	0.230 ± 0.026
Fr.9	0.110 ± 0.003	Fr.89	0.462 ± 0.036		
Fr.10	0.068 ± 0.003				
